# Non-Aqueous Sol-Gel Synthesis of FePt Nanoparticles in the Absence of In Situ Stabilizers

**DOI:** 10.3390/nano8050297

**Published:** 2018-05-03

**Authors:** Tobias Preller, Dirk Menzel, Saskia Knickmeier, Julian Cedric Porsiel, Bilal Temel, Georg Garnweitner

**Affiliations:** 1 Institute for Particle Technology, Technische Universität Braunschweig, Volkmaroder Straße 5, 38104 Braunschweig, Germany; t.preller@tu-braunschweig.de (T.P.); s.knickmeier@tu-braunschweig.de (S.K.); c.porsiel@tu-braunschweig.de (J.C.P.); b.temel@tu-braunschweig.de (B.T.); 2 Institute of Condensed Matter Physics, Technische Universität Braunschweig, Mendelssohnstraße 3, 38106 Braunschweig, Germany; d.menzel@tu-braunschweig.de; 3 Laboratory for Emerging Nanometrology (LENA), Technische Universität Braunschweig, 38106 Braunschweig, Germany

**Keywords:** non-aqueous, sol-gel synthesis, iron platinum, nanocrystals, superparamagnetic, fcc-FePt, hard magnetic, fct-FePt, Fe_3_Pt, FePt_3_

## Abstract

The synthesis of FePt nanocrystals is typically performed in an organic solvent at rather high temperatures, demanding the addition of the in situ stabilizers oleic acid and oleylamine to produce monomodal particles with well-defined morphologies. Replacing frequently-used solvents with organic media bearing functional moieties, the use of the stabilizers can be completely circumvented. In addition, various morphologies and sizes of the nanocrystals can be achieved by the choice of organic solvent. The kinetics of particle growth and the change in the magnetic behavior of the superparamagnetic FePt nanocrystals during the synthesis with a set of different solvents, as well as the resulting morphologies and stoichiometries of the nanoparticles were determined by powder X-ray diffraction (PXRD), small-angle X-ray scattering (SAXS), transmission electron microscopy (TEM), inductively coupled plasma optical emission spectroscopy (ICP-OES)/mass spectrometry (ICP-MS), and superconducting quantum interference device (SQUID) measurements. Furthermore, annealing of the as-prepared FePt nanoparticles led to the ordered L1_0_ phase and, thus, to hard magnetic materials with varying saturation magnetizations and magnetic coercivities.

## 1. Introduction

**Figure 1:**
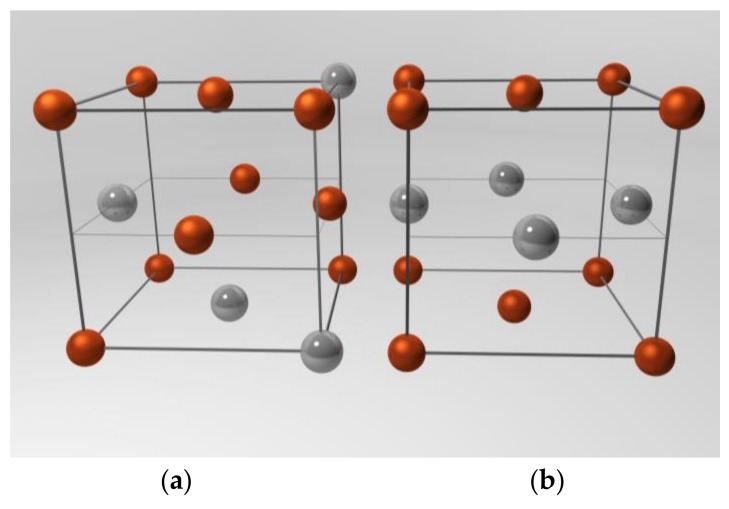
Illustration of (**a**) the unordered face-centered cubic (fcc) structure with randomly assembled iron (brown) and platinum (grey) atoms (arbitrarily chosen assembly) and (**b**) the ordered face-centered tetragonal (fct) phase with alternating layers of iron and platinum atoms (ordered L1_0_ phase).

Iron platinum (FePt) nanoparticles gained high scientific interest due to their large uniaxial magnetocrystalline anisotropy [1,2,3], high saturation magnetization and coercivity [4,5,6,7], as well as good chemical stability [1,8]. Those characteristics are promising with regard to potential applications, such as magnetic hyperthermia [4,9,10], biomedical imaging [11,12], ultrahigh density magnetic recording [13,14,15,16,17], or advanced permanent magnets [18,19,20,21]. Wet-chemical synthesis of nanosized FePt enables the preparation of monomodal nanoparticles with well-defined morphologies and particle sizes [1,16]. Thereby, the as-prepared Fe_x_Pt_1−x_ nanoparticles show disordered face-centered cubic (fcc) structures (Figure 1a) and, thus, superparamagnetic behavior. Thermal treatment of the particles at temperatures above 500 °C [1,22] leads to a rearrangement of the atoms in the unit cells (Figure 1b). An ordered face-centered tetragonal (fct) phase, the so-called L1_0_ phase and, thereby, a hard magnetic material emerges, if a ratio of approximately 1:1 of Fe and Pt atoms are present in the synthesized nanoparticles. However, higher variations in the Fe:Pt ratio result in the ordered L1_2_ phases of FePt_3_, Fe_3_Pt or mixtures of all three crystal structures [23].

Typically, iron platinum nanoparticles are prepared using chemical methods, following the polyol process, where diols or polyalcohols are used to reduce metal salts and prevent oxidation phenomena of the metal atoms [24]. Sun et al. [1] first reported the chemical reduction of platinum(II) acetylacetonate Pt(acac)_2_ by 1,2-hexadecanediol and thermal decomposition of iron(0) pentacarbonyl Fe(CO)_5_ for the synthesis of FePt nanoparticles with the in situ stabilizers oleic acid and oleylamine in the solvent octyl ether. Various amounts of the molecular precursors were used to generate monomodal iron platinum nanoparticles of defined stoichiometry and tunable particles sizes. Due to the toxicity of Fe(CO)_5_, alternative iron sources were later evaluated, such as iron(II) chloride [16,25] and iron(III) acetylacetonate Fe(acac)_3_ [26,27,28], also yielding narrow size-distributed FePt. Furthermore, nanoscale iron platinum was prepared using ethylene glycol, which serves as the organic medium and reducing agent simultaneously [29]. Additionally, several attempts for the direct synthesis of fct-FePt at temperatures of 300–400 °C or annealing of dissolved and dried precursors were reported [30,31,32].

In this work, we present the non-aqueous sol-gel synthesis of highly-crystalline FePt without the addition of in situ stabilizers. Therefore, we systematically tested the reaction of Fe(acac)_3_ and Pt(acac)_2_ in the solvents benzyl alcohol (BnOH), benzylamine (BnNH_2_), and triethylene glycol (TEG) for their ability to generate the desired binary alloy. In previous reports, we have already shown the successful synthesis of nanosized materials using the aforementioned solvents [33,34,35,36,37,38,39]. While the BnOH and TEG routes, in principle, follow the polyol process, the synthesis in benzylamine is performed in the presence of only amine moieties and, thus, is a novel preparation method of nanosized FePt. The syntheses were performed at elevated temperatures up to 200 °C under a nitrogen atmosphere. During the sol-gel process, we examined the particle growth as well as the magnetic behavior of the particles, and compared the kinetics and resulting properties to the formation of nanocrystalline FePt by a reference synthesis in the solvent benzyl ether (BnOBn) using the in situ stabilizers oleic acid and oleylamine. Thereby, the alternative synthetic routes led to superparamagnetic fcc-FePt with significantly increased saturation magnetization at comparable particle sizes. In addition, the as-prepared fcc-FePt nanoparticles were thermally treated at 800 °C and the resulting powders were analyzed by X-ray diffraction to evaluate the crystal structure and mean crystallite size, as well as by superconductive quantum interference device (SQUID) measurements to determine the magnetic behavior of the annealed particles. For each synthetic route, we obtained hard magnetic FePt nanoparticles of the ordered L1_0_ phase. Higher saturation magnetization or higher coercivity as compared to the fct-FePt of the reference synthesis could be achieved using the alternative synthetic routes. Thereby, the benzylamine route showed the highest saturation magnetization, while both the BnOH and TEG syntheses led to nanoparticles with a larger coercivity. Furthermore, we prepared nanocrystals of the ordered L1_2_ phases FePt_3_ and Fe_3_Pt through the precise adjustment of the Fe:Pt ratio in the synthesized nanoparticles.

## 2. Results and Discussion

**Figure 2:**
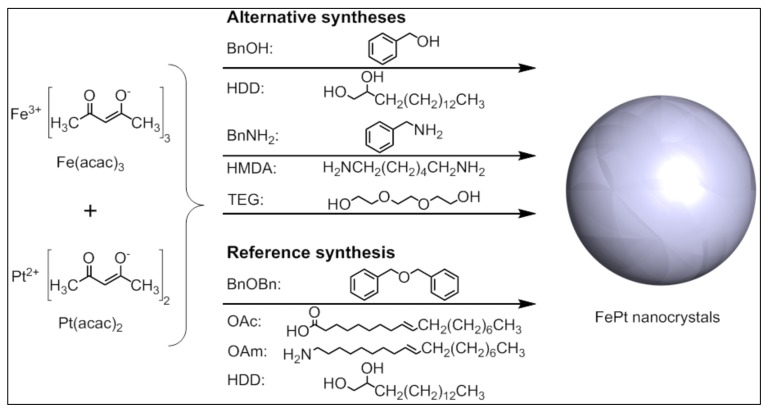
Schematic illustration of the investigated synthetic routes for fcc-FePt nanocrystals.

Three alternative non-aqueous sol-gel synthesis routes for the generation of superparamagnetic fcc-FePt nanocrystals with varying particle sizes and high saturation magnetization were tested without the addition of any in situ stabilizers using the solvents triethylene glycol, benzyl alcohol, and benzylamine, as well as the non-toxic molecular precursors iron(III) and platinum(II) acetylacetonate (Figure 2). To obtain the desired iron platinum alloy, the precursors were reduced by the addition of appropriate agents. First, we dissolved the chemical compounds at 70 °C and then heated the reaction mixture to the final temperature of 200 °C—or 185 °C in case of BnNH_2_—which is significantly lower than usually reported in the literature. As a control, we also generated iron platinum nanoparticles using the common polyol process including benzyl ether, oleic acid, and oleylamine.

During the syntheses, we observed a color transition from a reddish solution into the characteristic dark brownish medium, which is associated with particle growth. Henceforth, we started taking samples at 0, 1, 5, 15, and 45 min of reaction. The actual formation of the nanoparticles already occurred prior to the color shift, but is difficult to be precisely specified due to very low initial quantities of retrievable nanoparticles. The color transition of the reference BnOBn route, as also published earlier [40], was reached at a temperature of 175 °C approximately 45 min after complete dissolution of the precursors (Table A1). All three alternative synthetic routes showed the color shift around 150 °C and, thus, are about 15 min faster as compared to the reference synthesis.

**Figure 3:**
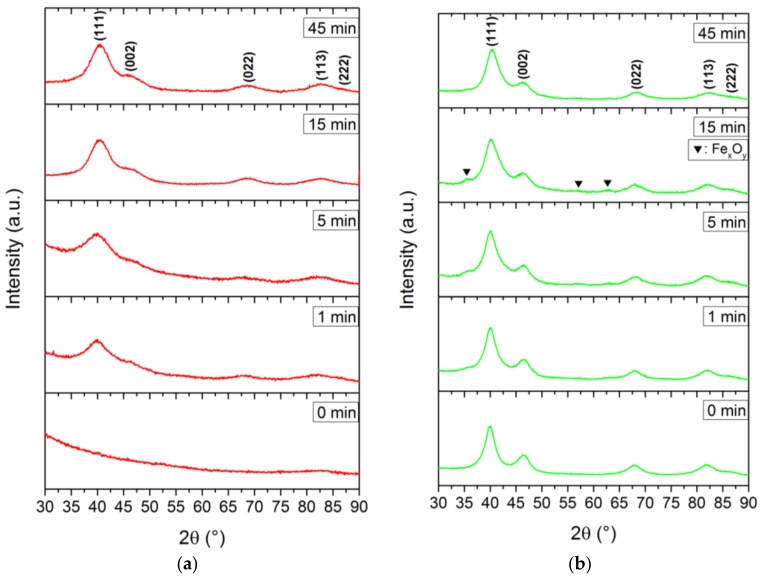
Diffraction patterns of the nanoparticles retrieved at 0, 1, 5, 15, and 45 min after the color transition of the reaction medium benzyl alcohol (**a**) and benzylamine (**b**).

**Figure A1:**
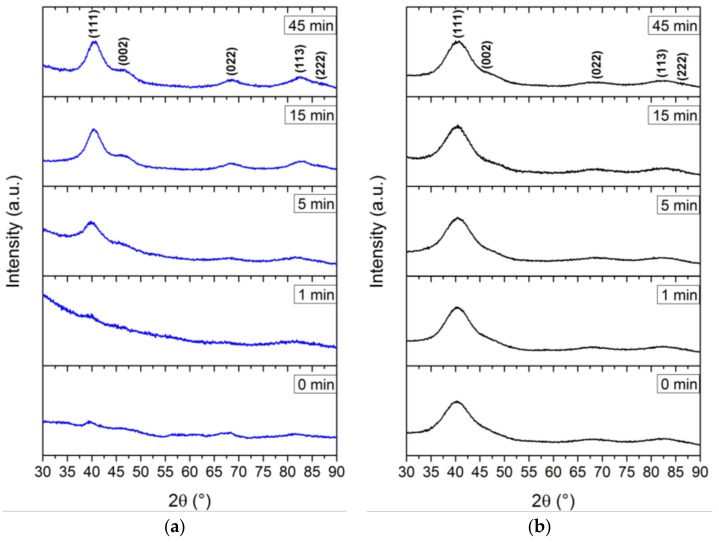
Diffraction patterns of the FePt nanoparticles synthesized in triethylene glycol (**a**) and benzyl ether (**b**).

All nanoparticles, except for the sample withdrawn at 0 min in BnOH, showed the specific signals of Fe_x_Pt_1−x_ with the characteristic (111) reflection around 40.0° 2θ. For fcc-FePt nanoparticles derived from the BnOH route, we observed a significant increase in their degree of crystallinity with progressing reaction times (Figure 3a). Similar diffraction patterns were obtained via the TEG synthesis (Figure A1a). We determined a less crystalline nature of the 0 and 1 min TEG samples probably evoked by low energy input with synthesis temperatures of 145 and 152 °C, respectively. After 5 min, a temperature of 178 °C was reached and the respective Fe_x_Pt_1−x_ signals evolved. The signal broadening is related to very small crystallite sizes. In contrast, the highest degree of crystallinity was achieved using the BnNH_2_ route, where the sample withdrawn at 0 min already revealed all characteristic reflections of fcc-FePt. Additional reflections at 35.5°, 57.0°, and 62.6°, however, evolved shortly after the color transition, which can be ascribed to the characteristic (113), (115), and (044) reflections of magnetite (ICSD 98-015-8741), respectively. We assume that the small amount of iron oxide emerges due to the oxygen-bearing acetylacetonate of the used precursors and small amounts of water dissolved in BnNH_2_ combined with an incomplete reduction of Fe^3+^ to Fe^0^. However, the impurities vanished in the further course of the synthesis and cannot be found in the final FePt nanoparticles. With increasing reaction time, minor changes in the degree of crystallization indicate only slightly increased crystallite sizes of the prepared iron platinum in benzylamine. This is observed in a similar fashion for the reference synthesis (Figure A1b).

**Figure 4:**
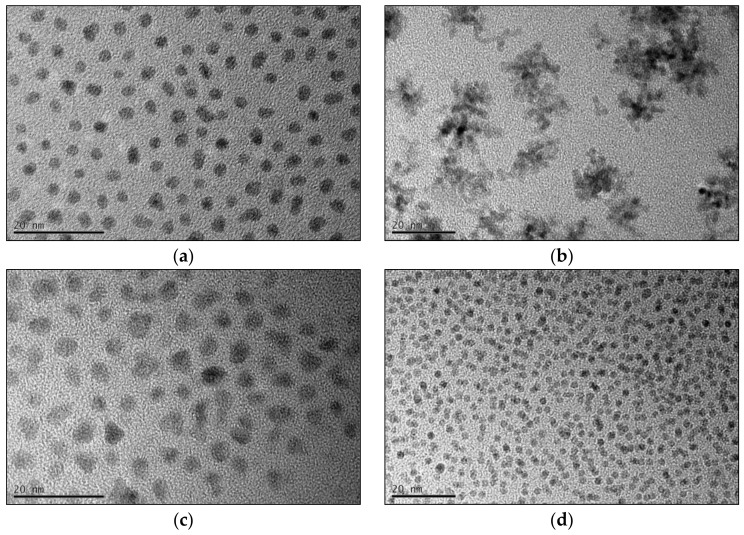
TEM images of the final prepared fcc-FePt nanoparticles using BnOH (**a**), BnNH_2_ (**b**), TEG (**c**), and BnOBn (**d**).

**Figure 5:**
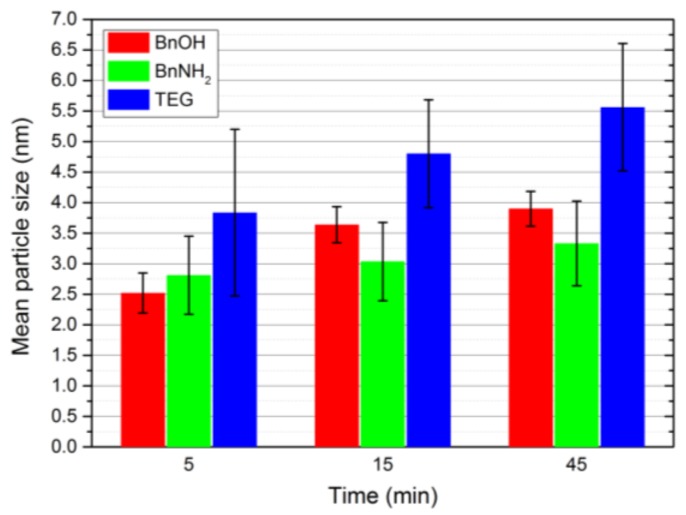
Diagram showing the mean particle size of the generated fcc-FePt nanoparticles for different reactions times.

The final fcc-FePt nanocrystals obtained by the BnOH synthesis feature a mean particle size of 3.9 nm with mostly spherical morphology (Figure 4a). In contrast, FePt created via the BnNH_2_ route led to larger particles in the beginning, but finally resulted in agglomerates about 20 nm in size consisting of 3.3 nm-sized crystallites (Figure 4b). Nanocrystals prepared in TEG continuously showed the highest particle sizes of all four performed syntheses. We achieved a mean size of 5.6 nm, with the nanocrystals exhibiting a more edgy shape (Figure 4c). Furthermore, the particles showed a higher hydrophilic character due to a larger extent of surface-bound solvent molecules. The control system led to significantly smaller particles of 2.3 nm with spherical shape (Figure 4d). The stoichiometry of the fcc-FePt nanoparticles was determined to between Fe_0.6_Pt_0.4_ and Fe_0.7_Pt_0.3_ for all systems (see Appendix C). Moreover, we monitored the increase in the particle size with progressing reaction times by the determination of the average sizes from TEM image analysis as well as SAXS measurements (Figure 5, see Appendix D for details). FePt nanoparticles prepared in BnOH showed a strong particle growth from 5 to 15 min. After 15 min, the desired synthesis temperature of 200 °C was reached and kept for 30 min. Thereby, a deceleration of the particle growth was observed. For the BnNH_2_ and TEG syntheses, the particle size constantly increased with reaction time. The highest standard deviation was observed for nanocrystals prepared in TEG.

**Figure 6:**
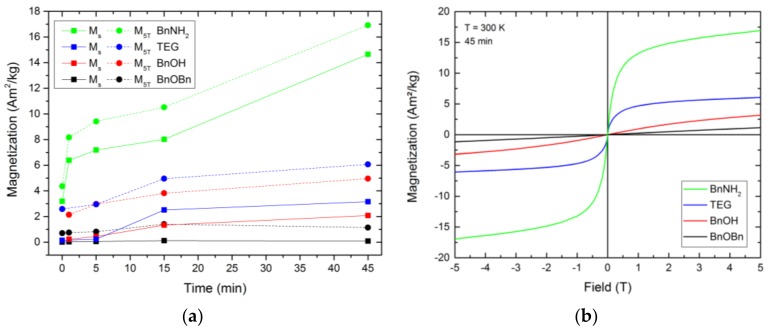
Saturation magnetization and magnetization at 5 T (**a**) as well as the superparamagnetic curves after 45 min reaction time (**b**) of the fcc-FePt nanoparticles derived from the reference synthesis (black) as well as alternative synthetic routes (red, blue, green).

All as-prepared fcc-FePt nanoparticles showed superparamagnetic behavior (Figure 6), which can be ascribed to their ferromagnetic iron atoms and small particle sizes below the critical diameter, where only one single magnetic domain exists in one particle [41,42]. In the literature, the maximum magnetization value is often used to describe the saturation magnetization M_s_ of the FePt nanoparticles. With our measurements, no saturation was reached with higher magnetic field strength within the measurement range. Instead, we observed a slightly increasing magnetization with the rising magnetic field, which we assume is evoked by an additional paramagnetic effect caused by the platinum atoms. Thus, we subtracted the paramagnetic influence using a linear fit on the magnetization values at 4 and 5 T and defined the intersection of this linear fit curve with the ordinate as the saturation magnetization. However, to show the highest measured magnetizations of the prepared particles and to allow comparison with previously published data, we also display the magnetization values at 5 T, M_5T_.

Each system showed rising magnetization values with increasing reaction time. The 0 min BnOH and 1 min TEG samples were not taken into account, due to very low sample quantities retrieved from the reaction mixture. However, we expect the magnetization values in a similar range as the adjacent samples. The lowest determined saturation magnetization values were observed for the nanosized FePt of the reference synthesis. After 45 min, the Fe_0.66_Pt_0.34_ nanoparticles resulted in M_s_ of 0.09 Am^2^kg^−1^ and M_5T_ of 1.14 Am^2^kg^−1^. In comparison, the final FePt nanoparticles synthesized in BnOH and TEG showed M_s_ of 2.08 and 3.16 Am^2^kg^−1^, as well as an M_5T_ of 4.96 and 6.07 Am^2^kg^−1^, respectively. The highest increase in the magnetic behavior was observed between 5 and 15 min. Thus, the magnetization strongly increases with enlarged particle sizes. The BnNH_2_ route led to unexpectedly high magnetization values. Although the actual particle size of the FePt crystals is smaller compared to the BnOH-FePt, a saturation magnetization of 14.65 as well as magnetization at 5 T of 16.92 Am^2^kg^−1^ were obtained. This can be attributed to the reduced interparticle distances in the formed agglomerates, which result in strongly increased magnetic dipole interactions and, thus, enhance the overall magnetic moment, coupled with the highest crystallinity observed for this system.

**Figure 7:**
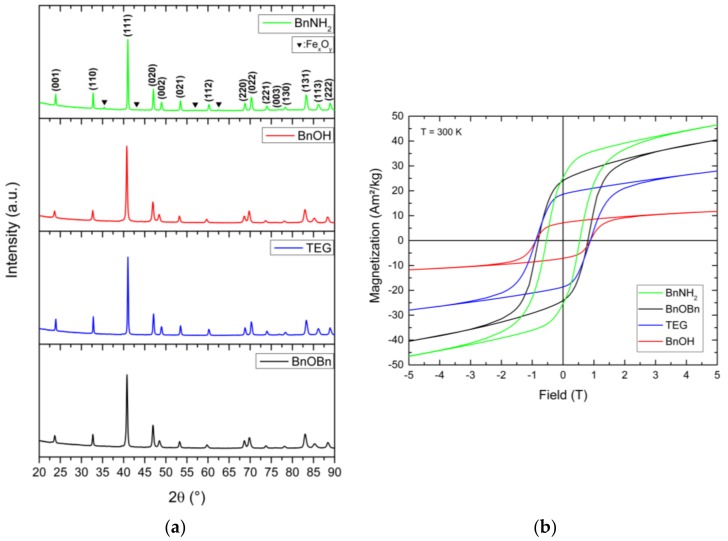
Diffraction patterns of the fct-FePt nanoparticles obtained after annealing at 800 °C (**a**) and superconductive quantum interference device (SQUID) measurements of the hard-magnetic properties of the generated ordered L1_0_ phase (**b**) for the different reaction systems (non-corrected curves).

Finally, the as-prepared face-centered cubic FePt nanoparticles were annealed at 800 °C under an argon atmosphere to induce the required rearrangement of the atoms in the unit cells in order to generate hard magnetic materials. The calcination process was performed for 2 h for particles prepared by the reference synthesis. For the alternative synthetic routes, calcination procedures of 2 h duration led to a mixture of various FePt phases. Phase-pure fct-FePt nanocrystals were obtained by extending the annealing time to 5 h (see Appendix E for BnOBn-FePt 5 h). Thereby, all characteristic reflections of the ordered L1_0_ phase (ICSD 98-016-8777) in the newly-generated materials could be identified (Figure 7a). For BnNH_2_-FePt, additional signals emerged that could be attributed to magnetite. The amount of iron oxide impurities in relation to the hard-magnetic iron platinum phase varied when using different washing agents for conditioning the fcc-FePt nanoparticles prior to the annealing process. As an example, the use of ethanol led to a 1:1 mixture of fct-FePt:Fe_3_O_4_ while chloroform resulted in 15% magnetite impurities, both determined by Rietveld refinement. In contrast, using oxygen-containing solvents for the synthesis, the formation of iron oxide could be entirely prevented. The crystallite sizes of the annealed fct-FePt nanoparticles were estimated using the Scherrer equation and amount to 37.5 nm for the BnOBn reference system (see Appendix F for a TEM image), 39.4 nm for BnOH, 63.8 nm for TEG, and 69.5 nm for BnNH_2_, analyzing the (111) reflection.

When exposed to an applied magnetic field, the annealed fct-FePt nanocrystals showed the characteristic magnetic hysteresis loops of hard magnetic materials (Figure 7b). FePt derived from BnOBn revealed high saturation magnetization and remanence M_r_ combined with a large coercivity H_c_ (Table 1). In contrast, the alternative synthesis with TEG led to hard magnetic FePt with lower M_s_ and M_r_, but increased coercivity. The same increase in H_c_ was observed for particles obtained by the BnOH route. However, the BnOH-FePt showed a marked decrease in saturation magnetization and remanence. For particles generated via the BnNH_2_ synthesis, we obtained the highest saturation magnetization. On the one hand, this is probably related to the enhanced sintering of particles in the formed agglomerates. The high annealing temperature of 800 °C evokes the formation of strong interparticle bonds between single crystals, which leads to the enlarged crystallite size. On the other hand, the additional soft magnetic iron oxide impurities magnetically interact with the hard magnetic FePt resulting in exchange coupling and, thus, in a high saturation magnetization combined with high coercivity.

**Figure 8:**
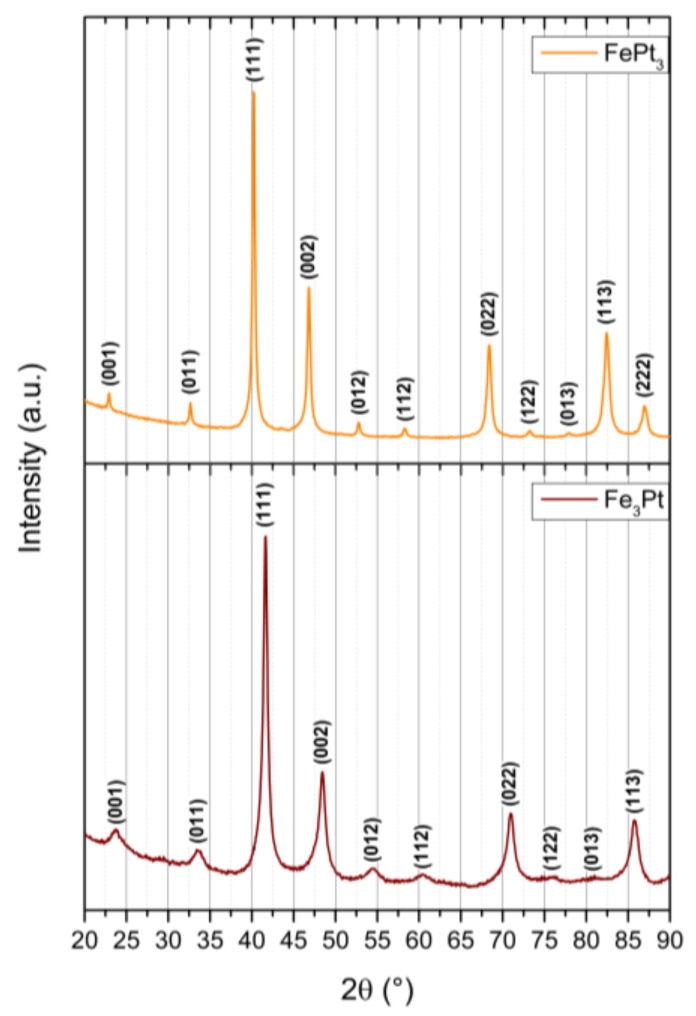
Diffraction patterns of the prepared FePt_3_ and Fe_3_Pt particles obtained by the BnOH route and annealed at 800 °C for 2 h.

**Figure 9:**
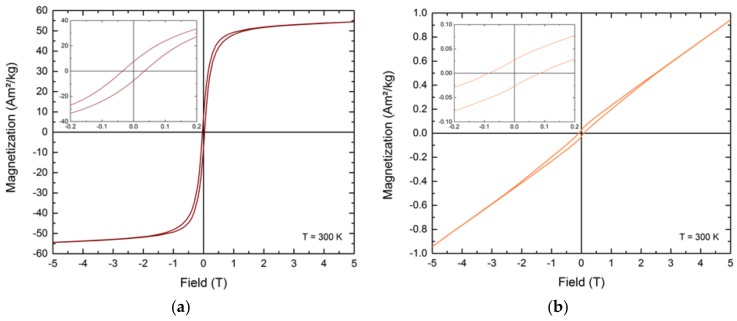
Magnetization curves of the predominantly ferromagnetic Fe_3_Pt (**a**) and paramagnetic FePt_3_ nanocrystals (**b**).

Apart from the hard magnetic iron platinum, we obtained the L1_2_ phases FePt_3_ and Fe_3_Pt through precise adjustments of the Fe:Pt precursor ratio during the non-aqueous sol-gel syntheses (Figure 8). While Fe_3_Pt possesses soft magnetic behavior combined with a small amount of paramagnetism from the unordered L1_2_ phase at room temperature, FePt_3_ predominantly exhibits a paramagnetic character associated by ferromagnetism of the minor unordered L1_2_ phase (Figure 9). Additionally, FePt_3_ shows two coexisting antiferromagnetic phases with Néel temperatures of T_N1_ = 160 K and T_N2_ = 100 K. Both L1_2_ phases gained increasing interest recently due to their exchange bias or exchange coupling effects in combination with fct-FePt [18,43,44].

## 3. Materials and Methods

### 3.1. Preparation of fcc- and fct-FePt Nanoparticles

Platinum(II) acetylacetonate (Pt(acac)_2_, 98%) was purchased from Acros Organics (Geel, Belgium). Iron(III) acetylacetonate (Fe(acac)_3_, 99.9% trace metal basis), benzyl ether (BnOBn, 98%), benzylamine (BnNH_2_, 99%), hexamethylenediamine (HMDA, 98%), 1,2-hexadecanediol (HDD, 90% technical grade), oleic acid (OAc, 90% technical grade), oleylamine (OAm, 70% technical grade), and triethylene glycol (TEG, 99%) were acquired from Sigma Aldrich (Darmstadt, Germany). Benzyl alcohol (BnOH, 99%) was obtained from Honeywell (Seelze, Germany).

The non-aqueous sol-gel synthesis of FePt nanoparticles was performed using an amount of 2 mmol each of the molecular precursors Fe(acac)_3_ and Pt(acac)_2_, for all reaction systems.

For the reference synthesis according to Akbari et al. [45], the metal organic precursors were mixed with 40 mL of the solvent benzyl ether and 10 mmol of 1,2-hexadecanediol, which was used to reduce the iron and platinum ions. Additionally, 20 mmol of the in situ stabilizers oleic acid and oleylamine were added.

For the alternative synthetic routes, Fe(acac)_3_ and Pt(acac)_2_ were mixed with 40 mL of BnOH, TEG or BnNH_2_. When using BnOH as solvent, reduction of the metal organic compounds was also induced adding 10 mmol of HDD. Triethylene glycol already serves as the solvent and reducing agent simultaneously and, therefore, no additional chemical compound was required.

For the synthesis in benzylamine, we replaced HDD with hexamethylenediamine (HMDA). Due to its lower reducing effect compared to the oxygen-containing agent, a higher amount of HMDA (20 mmol) was required.

The synthesis was performed under a nitrogen atmosphere in all cases. Thereby, the respective reaction mixture for each synthetic route was heated to a temperature of 70 °C within 20 min and held for another 10 min to dissolve the precursor compounds. Subsequently, the formed reddish solution was heated further until its color turned dark brown, which indicates increased particle growth. Henceforth, we started to measure the time and took samples at 0, 1, 5, 15, and 45 min. Due to the generally low yield of the samples at 0 and 1 min, and the fact that the particle growth would be drastically changed when removing higher quantities of the reaction mixture at the beginning of the color transition, each synthetic route was repeated only taking the 0 and 1 min samples. The desired reaction temperature of 200 °C was usually reached after 15 min and held for an additional 30 min. However, BnNH_2_ already boils at 185 °C, which was attained after 10 min. For comparability, we extended the synthesis for an additional 5 min.

The as-prepared FePt nanoparticles were conditioned by centrifuging using appropriate washing agents, and subsequently dried under vacuum. Finally, the obtained FePt nanoparticles were annealed at 800 °C for 2 or 5 h under an argon atmosphere.

For the preparation of Fe_3_Pt and FePt_3_ nanoparticles, the Fe:Pt ratio of the initial precursors was adjusted to 70:30 and 40:60, respectively, and the synthesis performed using the aforementioned BnOH route.

### 3.2. Characterization

Powder X-ray diffraction (PXRD) was carried out on vacuum-dried and annealed FePt samples to determine the crystal structure as well as crystallite size of the ordered L1_0_ and L1_2_ phases using Cu Kα radiation (Empyrean Cu LEF HR goniometer, Almelo, Netherlands) on a Si sample holder in a range between 20 and 90° 2θ with a step size of 0.05° (Empyrean series 2, PANalytical PIXcel-3D detector, Almelo, The Netherlands).

Transmission electron microscopy (TEM) images were obtained using a Tenai G2 F20 TMP device of FEI at 200 kV and 300 mesh holey carbon-coated copper grids (Plano GmbH, Wetzlar, Germany).

Whilst the particle size of the BnNH_2_ and TEG samples was determined by image analysis from overview TEM images, the particle sizes for the unannealed BnOH samples were determined by small-angle X-ray scattering (SAXS) using a SAXSess mc^2^ system of Anton Paar (Graz, Austria) utilizing Cu Kα radiation with a CCD detector. The experiments were carried out at room temperature within a 1 mm flow-through cuvette at a sample-to-detector distance of 309 mm and the obtained 1D patterns were normalized and corrected regarding the exposure time, transmission, instrumental background, and smearing effects. To gather structural information in real space, indirect Fourier transformations were performed using GIFT from the PCG software package [46] (see Appendix D).

ICP-OES was performed on an ICP-OES 715 ES from Varian (associated to Agilent, Santa Clara, CA, USA) for iron and ICP-MS on an ICP-MS 7700 from Agilent (Santa Clara, CA, USA) for platinum to examine the stoichiometry of the as-prepared fcc-FePt nanoparticles (Table A2).

**Figure A6:**
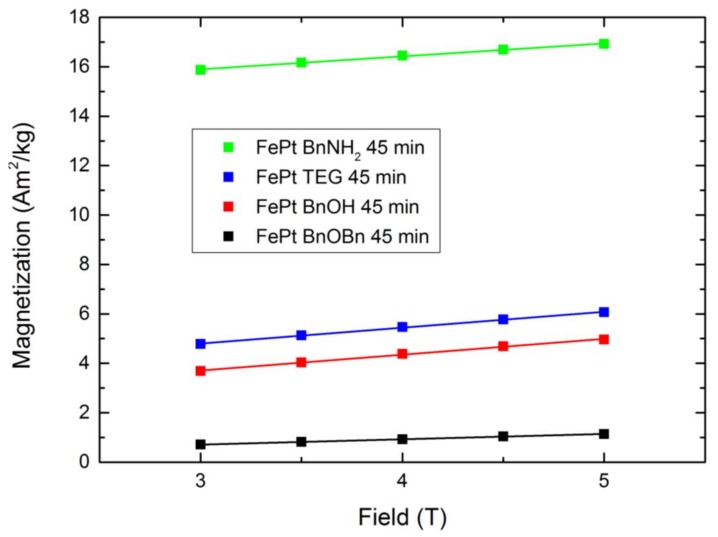
SQUID measurements of the final fcc-FePt nanoparticles of all systems.

To determine the magnetic behavior of the prepared FePt nanoparticles, superconductive quantum interference device (SQUID) measurements were performed on a vibrating sample magnetometer (Physical Properties Measurement System Dynacool, San Diego, CA, USA) from Quantum Design. No saturation magnetization could be directly determined from the measurements, due to additional paramagnetic effects from the included platinum atoms (Figure A6). Therefore, a linear fit on the magnetization values of 4 and 5 T was applied and the intersection with the ordinate defined as the saturation magnetization of the prepared FePt nanoparticles. To analyze the magnetic behavior of the samples obtained after 45 min of reaction and to obtain hysteresis curves of the calcined crystals, the FePt nanoparticles were embedded in epoxy resin and the diamagnetic moment of the matrix material was subtracted.

## 4. Conclusions

The synthesis of face-centered cubic iron platinum nanoparticles was established and investigated using the non-toxic and benign solvents benzyl alcohol, benzylamine, and triethylene glycol in the absence of in situ stabilizers. As control system, we used the widely-applied reference synthesis in benzyl ether, requiring the addition of hexadecanediol, oleic acid and oleylamine as stabilizers at comparable reaction conditions. Each route resulted in highly-uniform nanocrystals that could be stabilized individually, with the exception of the BnNH_2_ system, which resulted in small agglomerates. Kinetic investigations by withdrawal of samples during the syntheses showed an increase in the FePt particle size with progressing reaction time. The final as-prepared fcc-FePt nanoparticles are highly crystalline and phase-pure. The magnetic behavior was determined by SQUID measurements, where no magnetic saturation was observed during the measurement due to an additional paramagnetic influence caused by the included platinum atoms. By subtracting this effect applying a linear fit on the magnetization values of 4 and 5 T and determining the intersection of this line with the ordinate, a corrected saturation magnetization was calculated. In BnOH, a mean particle size of 3.9 nm, as well as a spherical morphology, was achieved. With a saturation magnetization of 2.08 Am^2^kg^−1^, a stronger magnetic response to an applied magnetic field was obtained compared to the FePt nanocrystals obtained from the reference system. The use of TEG led to a higher final particle size of 5.6 nm, as well as a more edgy shape of the nanocrystals. Due to the larger crystallite size, we observed an increased M_s_ of 3.16 Am^2^kg^−1^. The BnNH_2_ route, being a completely novel synthesis without any diol and polyalcohol species as present in the usual polyol synthesis, led to particles with a strongly increased M_s_ of 14.65 Am^2^kg^−1^. The observed spherical nanoparticles 3.3 nm in size showed a high tendency to form fractal agglomerates of about 20 nm in size, where the interparticle distances are reduced and, thus, significant magnetic dipole interactions arose. Additionally, the obtained fcc-FePt nanoparticles were annealed at 800 °C for 5 h and resulted in the ordered L1_0_ phase for all systems. The generated hard magnetic materials of the BnOH and TEG route showed an increased coercivity compared to fct-FePt derived from BnOBn. Furthermore, annealing of FePt nanocrystals of the BnNH_2_ synthesis exhibited the highest observed saturation magnetization of almost 35 Am^2^kg^−1^, probably resulting from exchange coupling due to the additional formation of small quantities of soft magnetic magnetite impurities. By variation of the precursor ratio, Fe_3_Pt and FePt_3_ were also obtained. The presented syntheses provide a versatile toolbox for the preparation of nanosized iron platinum particles with tailored hard magnetic properties.

## Figures and Tables

**Table 1:** Overview of the saturation magnetization M_s_, magnetization at 5 T M_5T_, remanence M_r_ and coercivity H_c_ of the prepared hard magnetic fct-FePt nanoparticles for the different reaction systems.

	M_s_ (Am^2^/kg)	M_5T_ (Am^2^/kg)	M_r_ (Am^2^/kg)	H_c_ (T)
BnOBn	30.02	40.50	24.09	0.80
BnOH	9.50	11.77	7.14	0.88
TEG	19.89	27.97	18.64	0.88
BnNH_2_	34.96	46.49	25.39	0.54

**Figure A2:**
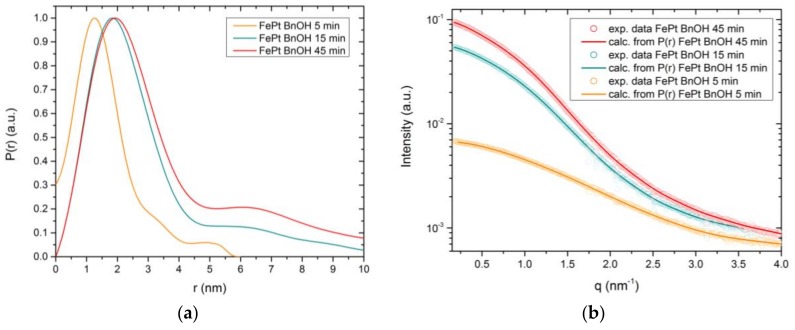
Pair-distance distribution functions (**a**) as well as scattering curves of the experimental data and calculations from P(r) (**b**) of the 5, 15, and 45 min samples of FePt-BnOH determined by small-angle X-ray scattering (SAXS). The scattering intensity is described as a function of the scattering vector q, with q = 4πsin(θ)/λ, λ as the corresponding wavelength of the X-ray source and scattering of the sample at an angle of 2θ. The P(r) function describes the relative frequency of all paired sets of interatomic distances within the system of length r and is used to gather dimensional and morphological information.

**Figure A3:**
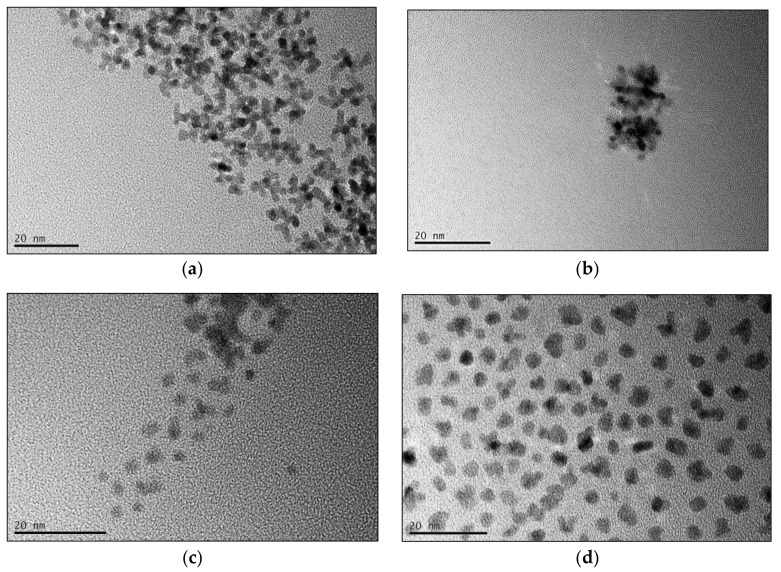
Transmission electron microscopy images of the 5 and 15 min samples of the benzylamine (**a**,**b**) and triethylene glycol (**c**,**d**) routes were used to analyze the particle growth during synthesis. Thereby, FePt particles derived by the BnNH_2_ route additionally reveal an increased formation of agglomerates. For TEG, an increase in the particle size, as well as a more edgy shape, could be observed.

**Figure A4:**
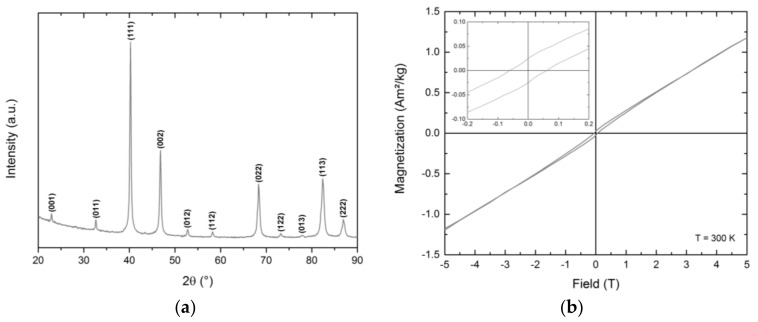
The diffraction pattern of BnOBn-FePt nanoparticles annealed at 800 °C for 5 h reveal the predominantly L1_2_ phase of FePt_3_ (**a**) and the magnetic characterization para- combined with ferromagnetism (**b**).

**Figure A5:**
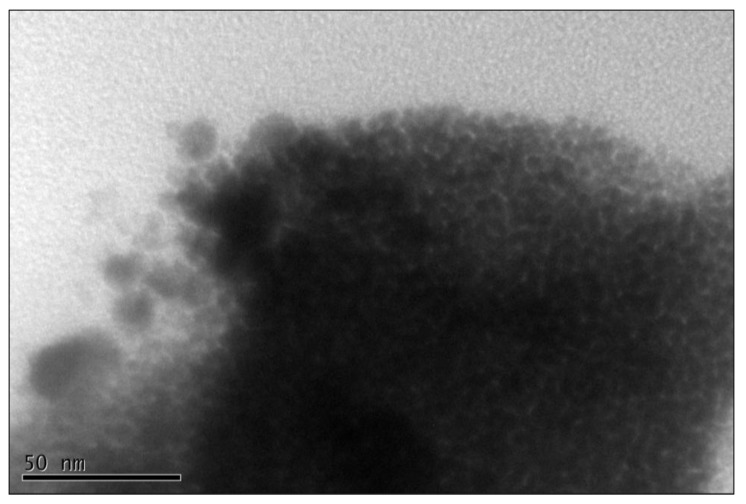
TEM image of fct-FePt of the reference synthesis annealed at 800 °C for 2 h.

## Appendix A

During the course of the syntheses, the temperature of the reaction medium was recorded. We observed the color transition of the reference synthesis at much higher temperatures compared to the alternative synthetic routes. The temperature rose for 15 min, except of the BnNH_2_ route, and then was held constant for 30 min.

**Table A1:** Listing of the temperature records during sampling.

	0 min	1 min	5 min	15 min	45 min
BnOBn	175 °C	177 °C	190 °C	200 °C	200 °C
BnOH	153 °C	158 °C	170 °C	200 °C	200 °C
TEG	145 °C	152 °C	178 °C	200 °C	200 °C
BnNH_2_	156 °C	159 °C	170 °C	185 °C	185 °C

## Appendix B

The diffraction patterns of the FePt nanocrystals derived by the TEG route and reference synthesis are shown in Figure A1.

## Appendix C

The amounts of iron and platinum present in the as-prepared fcc-FePt nanoparticles were analyzed by ICP-OES and ICP-MS, respectively (Table A2). Those measurements were used to determine the stoichiometry of the nanocrystals to between Fe_0.6_Pt_0.4_ and Fe_0.7_Pt_0.3_. It is known from the literature that as-prepared nanoparticles with slightly increased iron content preferentially result in the ordered L1_0_ phase after annealing [23,28].

**Table A2:** Stoichiometry of the fcc-FePt nanocrystals as determined by ICP-OES (Fe content) and ICP-MS (Pt content).

	Composition
BnOBn	Fe_0.66_Pt_0.34_
BnOH	Fe_0.66_Pt_0.34_
TEG	Fe_0.67_Pt_0.33_
BnNH_2_	Fe_0.69_Pt_0.31_

## Appendix D

For the determination of the particle growth during the syntheses, we used both transmission electron microscopy, as well as small-angle X-ray scattering on the 5, 15, and 45 min samples of the alternative synthetic routes. SAXS analysis of the FePt particles prepared in BnOH resulted in evaluable scattering curves and pair-distance distribution functions P(r), showing increasing mean particle sizes of 2.5, 3.6, and 3.9 nm, respectively (Figure A2). However, the sizes of the BnNH_2_ and TEG samples could not be examined due to fast particle precipitation. Therefore, we additionally used TEM images to characterize the size of the nanocrystals (Figure A3). For the 45 min sample of BnOH, both characterization techniques were performed, leading to mostly the same mean particle size with 3.8 nm being determined by TEM and 3.9 nm by SAXS.

## Appendix E

The as-prepared FePt nanoparticles of the reference synthesis were also annealed at 800 °C for 5 h, but resulted mainly in the L1_2_ phase FePt_3_ (Figure A4). Magnetic characterization reveals a combination of para- and ferromagnetism and, therefore, a partially-ordered phase.

## Appendix F

The fct-FePt crystals of the reference synthesis annealed at 800 °C for 2 h were analyzed by transmission electron microscopy (Figure A5).

## Appendix G

The originally-measured magnetization values of the fcc-FePt nanoparticles of all systems were determined by SQUID with an applied magnetic field between 3 and 5 T (Figure A6).
